# Supplementary material for: Rapid incorporation of Favipiravir by the fast and permissive viral RNA polymerase complex results in SARS-CoV-2 lethal mutagenesis
Source: Nat Commun. 2020 Sep 17;11:4682. doi: 10.1038/s41467-020-18463-z (PMC7499305; doi:10.1038/s41467-020-18463-z)
Supplement: Supplementary file 1 — Supplementary Information [file 41467_2020_18463_MOESM1_ESM.pdf]

Supplementary Information

Rapid incorporation of Favipiravir by the fast and permissive viral RNA polymerase complex results in SARS-CoV-2 lethal mutagenesis

Shannon et al.

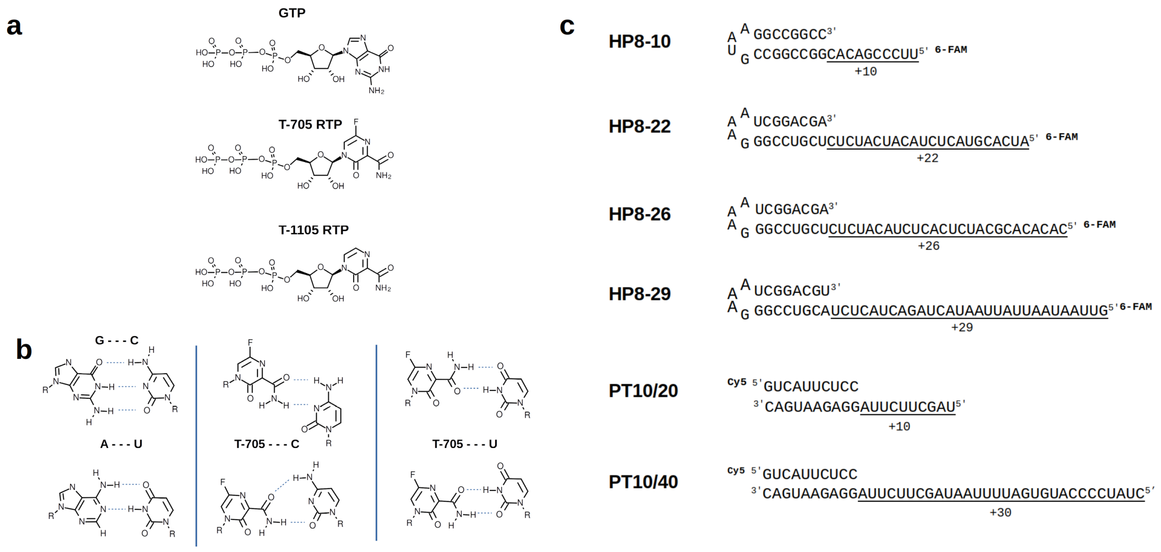

Supplementary Figure 1. Nucleoside analogues and RNA substrates. a, Chemical structures of the nucleotide guanosine 5'-triphosphate (GTP), the chain terminator T-705 ribofuranosyl 5'-triphosphate (T-705-RTP), and its non-fluorinated analogue (T-1105-RTP). **b,** Schematics of standard base pairing guanine – cytidine and adenine – uridine (left) versus ambiguous base pairing of Favipiravir (T-705) opposite to a cytidine (central) or a Uridine (right) RNA template. **c,** Hairpin RNAs (HP) are labelled at the 5' end with 6-Carboxyfluorescein (6-FAM). Nomenclature of HP8-X refers to the length of the duplex stem (8) and length of the overhanging, single-stranded templating strand for extension (X). The primer of the primer-template (PT) substrates are 5' labelled with cyanine-5 (Cy5). Numbers refer to the length of the primer and template strand respectively.

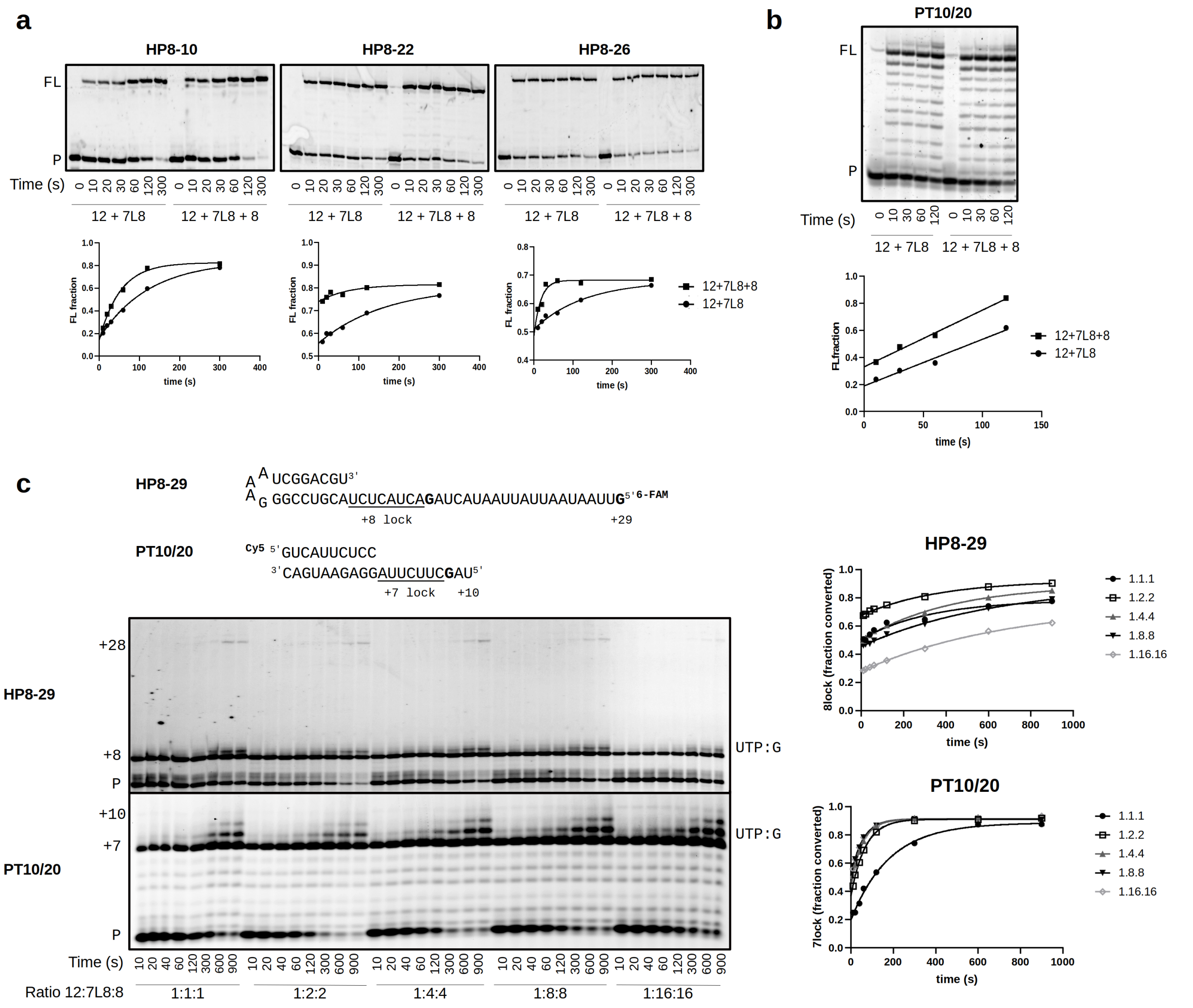

Supplementary Figure 2. Comparison of activity of nsp12:7L8 and nsp12:7L8:8 complexes. a, Extension of varied HP and **b,** PT substrate by either the nsp12+nsp7L8 complex (1:10 molar ratio) or nsp12+7L8+8 complex (1:10:5 molar ratio). Graphs show the fraction of full-length (FL) product produced at each timepoint relative to the total RNA. **c,** Extension of HP and PT substrates by the nsp12:7L8:8 complex at different molar ratios (as indicated). Reactions were run with run with 50 µM final concentration of ATP, UTP and GTP (omitting CTP) to form +8 and +7 stalled complexes for the HP and PT substrates respectively. Read-through products resulting from a nucleotide UTP misincorporation event against the templated G are seen for both substrates (UTP:G). Graphs show the fraction of stalled +7/+8 product relative to the amount of total RNA. Source data are provided as a Source Data file.

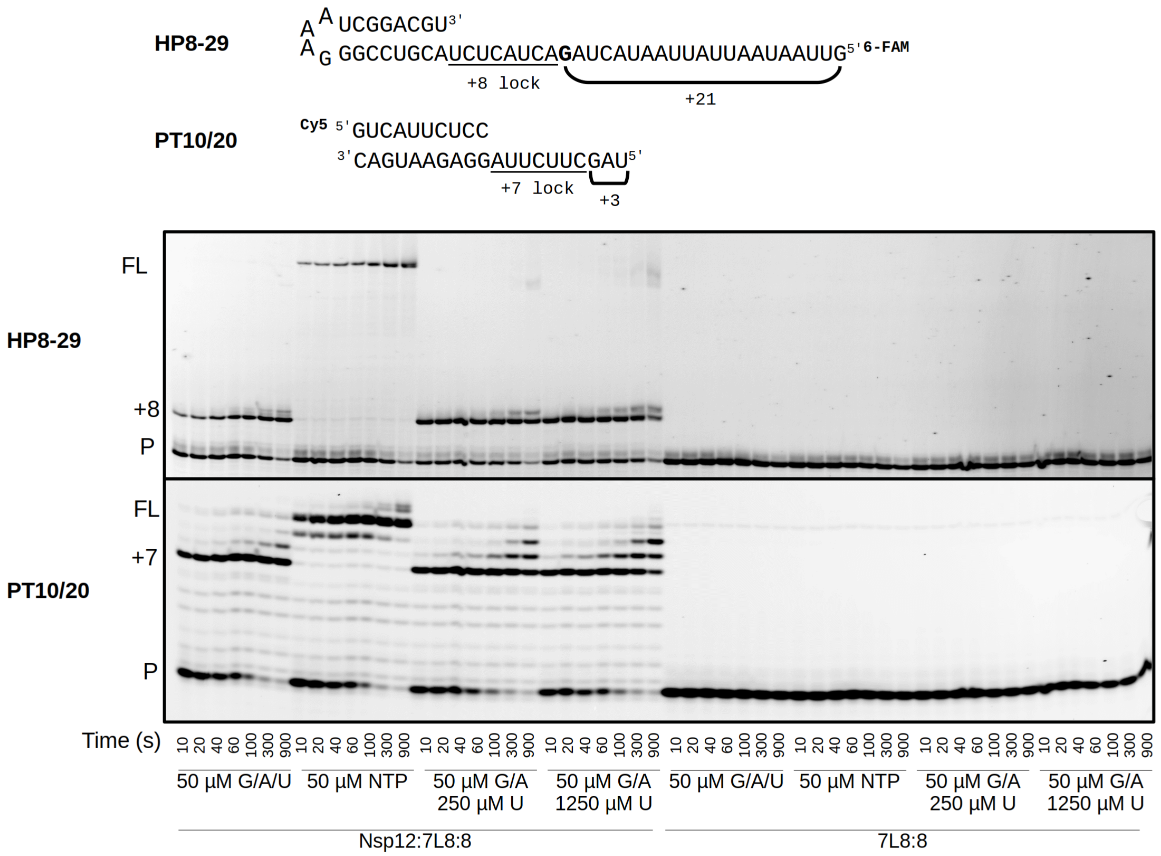

Supplementary Figure 3. Elongation with varied NTP combinations in the presence and absence of nsp12. Extension of HP (top) and PT (bottom) substrates by either the nsp12:7L8:8 complex or with nsp7L8:8 alone. Reactions were initiated with varied NTP conditions, either all four NTPs, or with GTP, ATP and UTP only (omitting CTP), with UTP concentration varied 5-fold (50, 250 and 1250 µM as indicated). Primer (P), +7/8 lock species and full-length (FL) products shown for both substrates. No primer elongation is observed in the absence of nsp12.

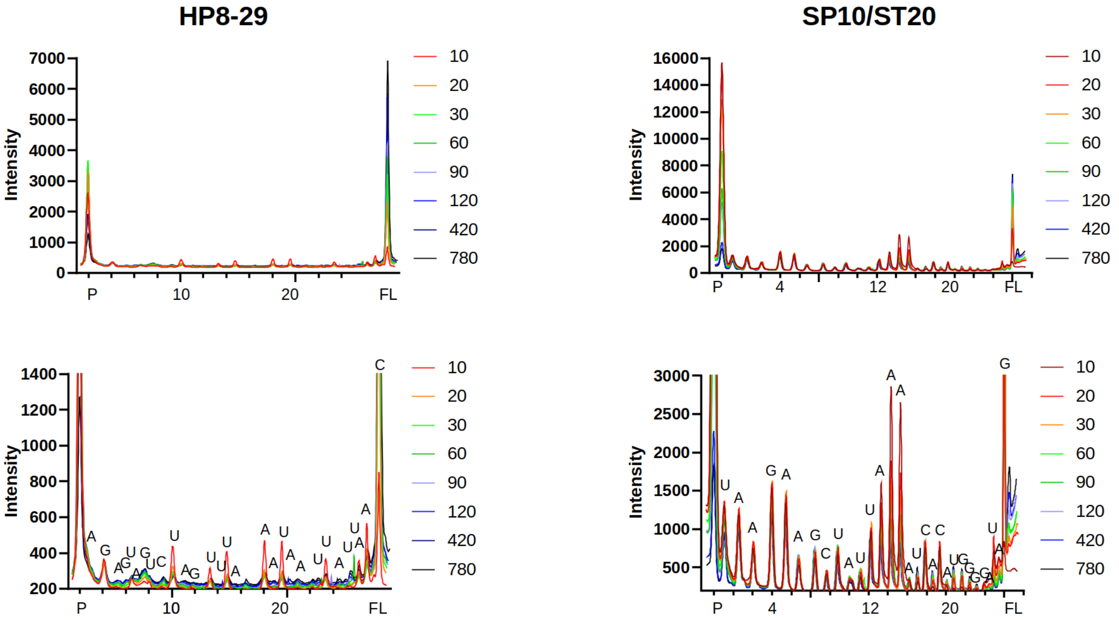

Supplementary Figure 4. Intermediate species over time shown in Fig. 1. Lane profiles of manual quench experiments performed with HP8-29 and PT10/40 substrates were obtained using ImageJ (Fiji). Peaks were normalized along the x-axis, and are shown from primer (P) to full-length (FL) for each substrate. Top panels show raw lane intensity for each substrate. Bottom panels are scaled 5X for relative peak analysis, with band assignments shown. Colors represent data taken from each timepoint in seconds. Source data are provided as a Source Data file.

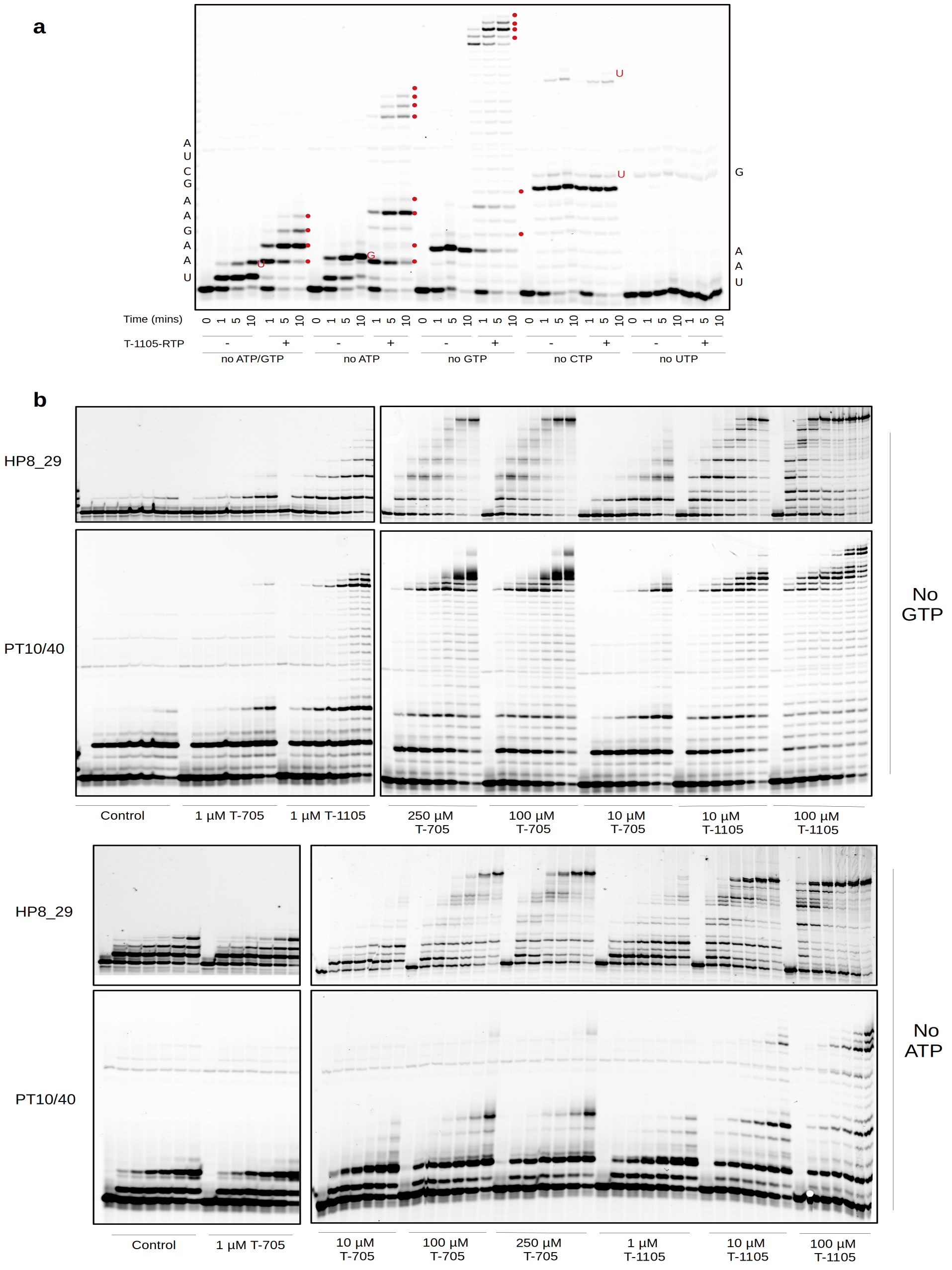

Supplementary Figure 5. Nsp12:7L8:8 incorporation of nucleoside analogues into RNA.
a, Extension of PT10/40 substrate in the presence or absence of T-1105-RTP (10 µM) with either one or two NTPs omitted and 50 µM of other NTPs. Incorporation of analogues shown with red dots. Nucleotides on the side of gel indicate correct nucleotide incorporation for first 10 positions, and nucleotides in red on gel indicate incorporation of incorrect nucleotides. **b,** Complete data for Fig. 2C. Incorporation of varied concentrations of nucleoside analogues T-705 and T1105 with different RNA substrates in the absence of GTP (top) or ATP (bottom). Time course for all series is 0, 10 s, 20 s, 40 s, 60 s, 2 mins and 5 mins.

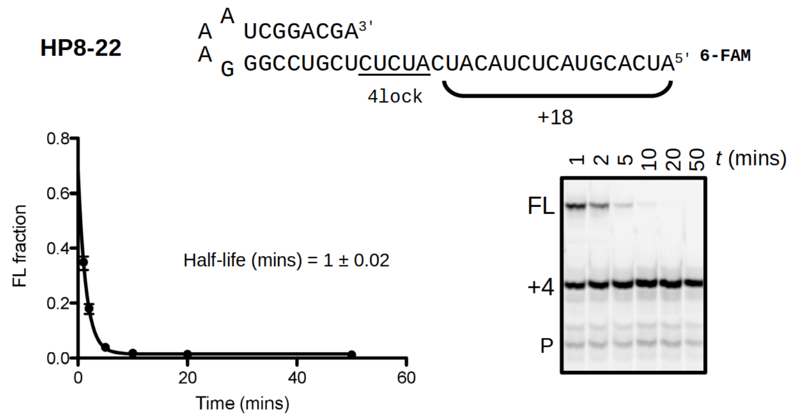

Supplementary Figure 6. Formation of a stable elongation complex on HP RNA. Nsp12:7L8:8 complexes at varied ratios (1:1:1 – 1:10:10) were incubated with HP RNA and 50 µM GTP and ATP for 5 mins to promote formation of 4nt-lock, and diluted 1:2 in high salt to prevent RNA rebinding. Reactions were chased at indicated timepoints with 50 µM all NTPs. Chase reactions were quenched after 30 sec and analyzed by gel electrophoresis. Stability half-life was calculated from the ratio of full-length (FL) product produced relative to the total amount of +4-lock + FL product at each timepoint. No significant difference in ratios was observed, and thus data was analysed as the average FL product produced for all ratios (n=6), with S.E shown. 95% C.I for half-life 0.93 – 1.08. Source data are provided as a Source Data file.

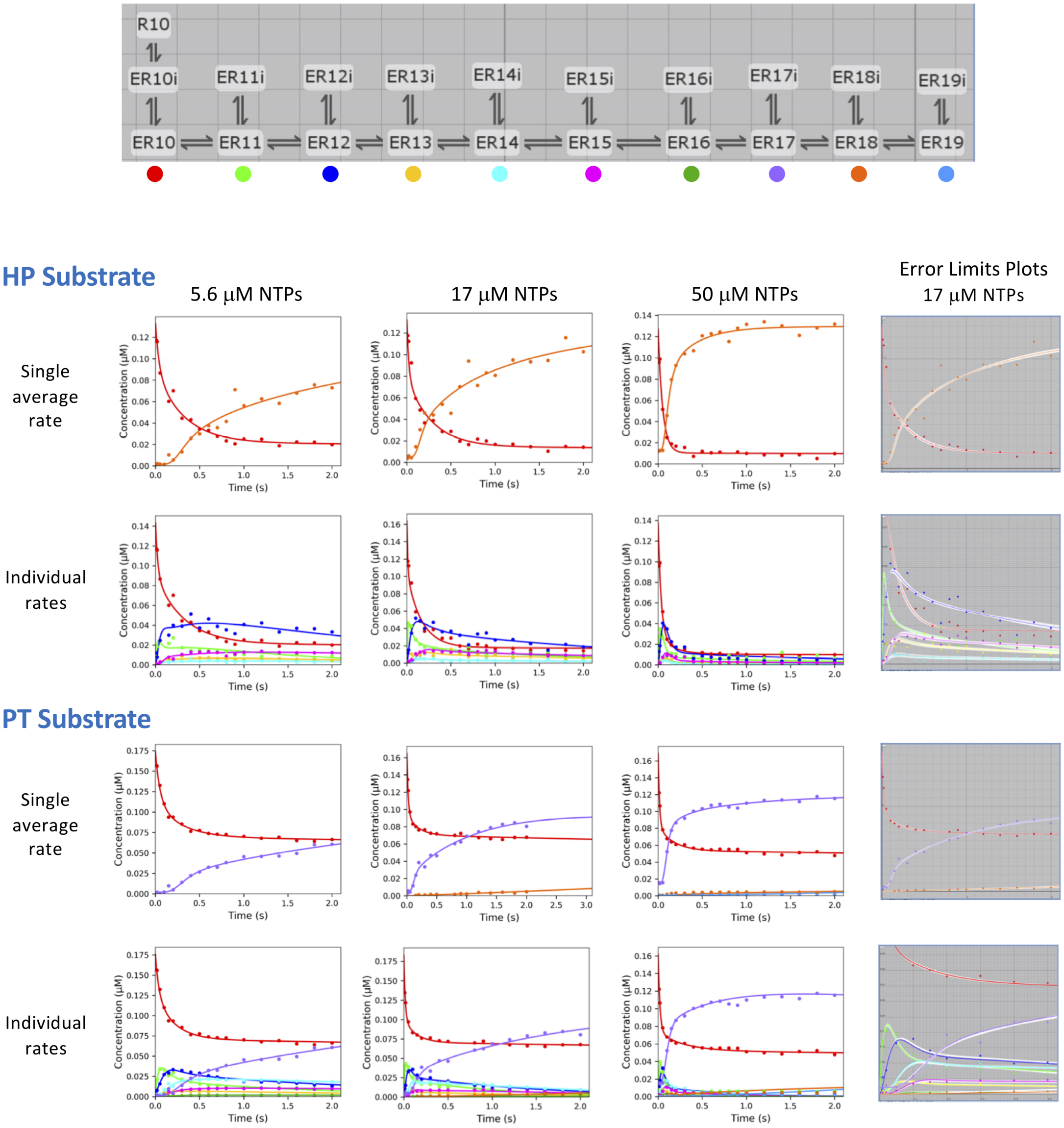

Supplementary Figure 7. Kinetic modeling of pre-steady state quench-flow data. Elongation kinetics were analyzed using KinTek Explorer with a model for sequential addition steps wherein in every elongation state was in an equilibrium with an off-pathway inactive species to account for the large amounts of intermediate species observed. Initial concentrations of the ER10 species reflect pre-assembled polymerase-RNA complexes capable of immediate burst phase elongation, and the R10 species reflects unbound RNA that is slowly depleted in the reaction, leading to the slow final phase of full-length product formation. An average rate was determined by making all elongation steps equal and considering only the primer and full-length product, while the individual rates analysis allowed each nucleotide addition step to vary independently. The inactivation/reactivation steps were always fit to a single common set of rates for all species. The plot on the far right show the 17 uM NTP results where each species curve has a white border that represents its standard error limits from kinetics analysis. See Supplementary Table 1 for complete results, individual parameter errors, and confidence intervals arising from the kinetic fitting.

Optimized iterative synthesis of T-1105-RDP and -RTP

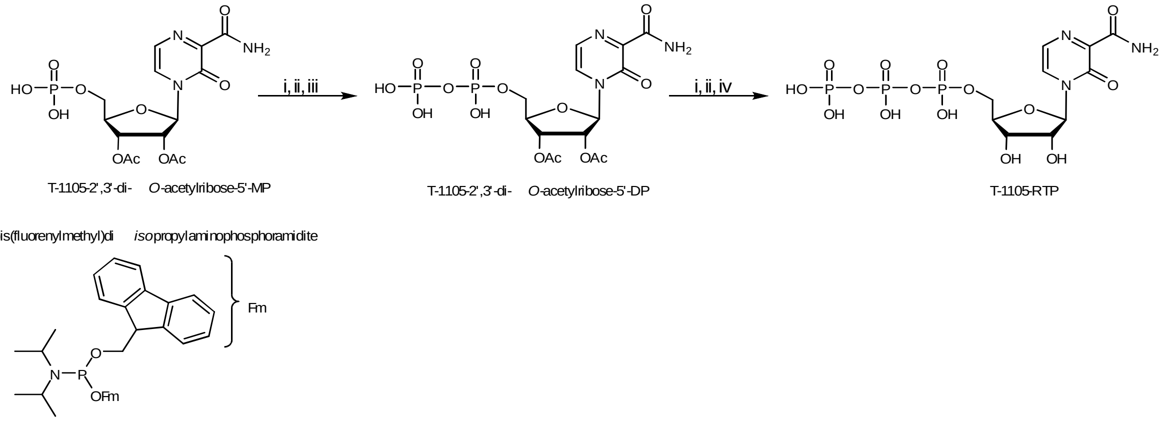

Supplementary Figure 8. T-1105-RTP was synthesized from T-1105-2‘,3‘-di-*O*-acetylribose-5‘-monophosphate via T-1105-2‘,3‘-di-*O*-acetylribose-5‘-diphosphate following the established route (Huchting, J. *et al*. (2018) J. Med. Chem. 61, 6193–6210. Acetyl-groups were kept until the final TP-synthesis to overcome low solubility. Reactions were performed under anhydrous conditions and at room temperature if not indicated otherwise.

Preparation of T-1105-2‘,3‘-di-*O*-acetylribose-5‘-diphosphate: **i)** T-1105-2‘,3‘-di-*O*-acetylribose-5‘-monophosphate (0.11 mmol, $TEAH^+$-salt) was dissolved in acetonitrile and bis(fluorenylmethyl)di*iso*propylaminophosphoramidite (1.6 eq., dissolved in dichloromethane) was added, followed by dropwise addition of dicyanoimidazole (1.6 eq., 0.25 M solution in acetonitrile). The reaction progress was monitored via TLC (solid phase: silica, eluent: acetonitrile/ water 7:1). After stirring for 20 min, *tert*butylhydroperoxide (3 eq., 5.5 M solution in decane) was added and the mix was stirred for another 10 min before all volatiles were evaporated. **ii)** The crude product was taken up in acetonitrile containing 10% triethylamine and stirred for 10 min, followed by evaporation and RP-flash chromatography (Interchim PuriFlash, prepacked C18-modified silica column 16 g, 15 mL min^{-1}, min 0-3 100% water, min 3-20 linear gradient to 100% acetonitrile). **iii)** Product-containing fractions were pooled, evaporated, dissolved in acetonitrile containing 20% triethylamine, stirred for 16 h, and again evaporated. T-1105-2‘,3‘-di-*O*-acetylribose-5‘-diphosphate was isolated in 91% yield (0.097 mmol) *via* RP-flash chromatography (Interchim PuriFlash, prepacked C18-modified silica column 16 g, 15 mL min^{-1}, min 0-8 100% water, min 8-20 linear gradient to 100% acetonitrile).

Preparation of T-1105-ribose-5‘-triphosphate: Starting from T-1105-2‘,3‘-di-*O*-acetylribose-5‘-diphosphate (0.02 mmol), steps **i** and **ii** were performed as described above. **iv)** Then, product-containing fractions were pooled, evaporated, dissolved in water containing 10% methanol and 20% triethylamine, stirred for 4.5 h at room temperature to 50°C (water bath), and again evaporated. T-1105-ribose-5‘-triphosphate was isolated in 46% yield (0.0092 mmol) *via* RP-flash chromatography (Interchim PuriFlash, prepacked C18-modified silica column 16 g, 15 mL min^{-1}, min 0-10 100% water, min 10-12 linear gradient to 100% acetonitrile). Finally, cations were exchanged to Na^+ on DOWEX50WX8 cation exchange resin.

Single Average Rate Analysis						
	Primer-Template (PT10/20)			Hairpin (HP8-29)		
	5 μM	16.7 μM	50 μM	5 μM	16.7 μM	50 μM
k_{pol}	18–20<br>*18.7±0.9*	45–51<br>*48±2*	64–68<br>*66.0±1.3*	21–28<br>*23±3*	40–55<br>*46±6*	90–103<br>*95±8*
k_{inact}	3.8–4.8<br>*4.2±0.4*	8.8–11<br>*9.7±0.8*	3.1–3.9<br>*3.5±0.2*	2.6–5.0<br>*3.5±0.9*	3.9–6.9<br>*4.9±1.5*	4.2–7.4<br>*5.3±1.5*
k_{react}	0.5–0.7<br>*0.60±0.09*	1.7–2.1<br>*1.83±0.14*	0.75–1.2<br>*0.93±0.17*	0.5–1.0<br>*0.8±0.2*	0.9–1.7<br>*1.2±0.4*	3.1–5.1<br>*3.9±1.0*
$k_{UTP:G*}$	–	0.16–0.25<br>*0.20±0.04*	0.12–0.16<br>*0.14±0.01*			
UTP:G* vs Average	–	1/240	1/470			

Individual Steps Rate Analysis							
Primer-Template (PT10/20)				Hairpin (HP8-29)			
NTP : Base	5 μM	16.7 μM	50 μM	NTP : Base	5 μM	16.7 μM	50 μM
**+1** UTP:A	25.0±1.0	65±2	51.0±1.2	**+1** ATP:U	38±3	104±8	29.4±1.3
**+2** ATP:U	12.6±0.7	34.0±1.6	39.8±1.4	**+2** GTP:C	37±6	23.8±1.7	46±4
**+3** ATP:U	8.7±0.7	22.2±1.1	39.9±1.5	**+3** ATP:U	9.0±1.5	8.9±0.6	25±2
**+4** GTP:C	57.6±0.8	84±9	151±15	**+4** GTP:C	53±12	30±3	140±40
**+5** ATP:U	12.4±1.7	20.6±1.1	45±2	**+5** UTP:A	80±30	80±20	230±110
**+6** ATP:U	21±3	39±3	70±5	**+6** ATP:U	21±4	19±2	76±13
**+7** GTP:C	120±40	280±130	210±40	**+7** GTP:C	Not fit	Not fit	Not fit
**+8** UTP:G*	–	0.21±0.04	0.23±0.03	**+8** UTP:A	Not fit	Not fit	Not fit
**+9** UTP:A	–		1.6±0.8				
k_{inact}	6.5±1.1	8.0±0.6	3.9±0.3	k_{inact}	8±2	3.2±0.5	2.6±0.5
k_{react}	0.63±0.06	1.2±0.1	1.7±0.2	k_{react}	0.8±0.2	0.57±0.13	0.4±0.2
UTP:G* vs UTP:A	–	1/310	1/220				

Supplementary Table 1. Pre steady-state elongation rates. Values (s^{-1}) obtained from fitting the kinetic model shown in Supplementary Figure 7 to the EDTA quench data with KinTek Explorer. The single average rate analysis lists both 90% confidence intervals and optimal values with standard errors, while the individual steps analysis lists only optimal values. For the HP substrate, the +7 and +8 product bands could not be definitively identified in gels. Values in red and with * represent putative UTP:G mismatch events observed on the PT substrate and the ratios of these rates to either the single average rate or to a cognate UTP:A addition.

FwpQE30PIPE	CACCATTAATAAAAGCTTAATTAGCTGAGCTTGG
RVpQE30PIPE	CATAGTTAATTTCTCCTCTTTAATG
FwpQE30nsp8	CGCGGATCCGAAAACCTGTACTTCCAGGGTGCTATTGCTTCAGAATTTAGTTCTTTACC
RvpQE30nsp8	GGCCCCTGCAGTTATTACTGTAGTTTAACAGCTGAGTTGGC
FwpJ404C8His	CCTGTACTTCCAGGGTCATCACCACCATCACCATCACCATTAACTCGAGCCCC
RvpJ404C8His	GGGGCTCGAGTTAATGGTGATGGTGATGGTGGTGATGACCCTGGAAGTACAGG

Supplementary Table 2. Specific primers used for cloning. Existing constructs[1] were modified using primers FwpQE30PIPE and RvpQE30PIPE for nsp7L8 constructs (for subcloning using PIPE into pQE30 vector), FwpQE30nsp8 and RvpQE30nsp8 for generation of N-terminally His tagged nsp8 using BamHI and PstI restriction sites and FwpJ404C8His and RvpJ404C8His for modification of C-terminal Strep tag to 8-histidine tag for nsp12.

ATGAGCGCTGATGCATCCACCTTCCTGAACAGAGTCTGCGGCGTGAGCGCAGCACGTTTGACCCCGTGCGGTACGG
GTACGTCGACCGATGTAGTGTACCGCGCGTTCGACATCTACAACGAGAAGGTTGCGGGCTTCGCCAAGTTCCTGAA
AACCAACTGTTGCCGTTTCCAAGAGAAGGACGAAGAGGGCAACCTGCTGGACTCCTATTTTGTTGTTAAACGTCAC
ACGATGAGCAATTACCAACACGAGGAAACCATTTACAATCTGGTTAAAGACTGTCCAGCGGTTGCCGTACACGAC
TTCTTTAAGTTCCGCGTCGACGGCGATATGGTGCCGCACATTAGCCGTCAGCGTCTGACGAAATACACCATGGCTG
ATTTGGTTTACGCCCTGCGCCACTTCGACGAAGGCAACTGCGACACGCTGAAGGAGATTCTGGTGACGTACAATTG
CTGCGACGACGATTACTTCAACAAGAAAGACTGGTACGACTTCGTGGAGAATCCGGACATTTTGCGTGTTTACGCC
AATTTGGGCGAGCGCGTGCGTCAAAGCTTGCTGAAAACCGTGCAGTTTTGCGATGCCATGCGTGATGCGGGTATCG
TCGGCGTGCTGACCCTGGATAACCAGGATCTGAACGGTAACTGGTATGATTTTGGTGATTTCGTTCAGGTGGCACC
GGGTTGTGGTGTGCCTATCGTTGACTCTTATTACAGCCTGCTGATGCCGATTCTGACGCTGACCCGTGCGCTGGCTG
CGGAGAGCCACATGGACGCCGATCTGGCAAAGCCGCTGATCAAGTGGGACCTGCTGAAATATGATTTTACCGAAG
AGCGTCTGTGTCTGTTTGATCGTTACTTCAAGTACTGGGATCAAACCTACCACCCGAATTGTATCAACTGTCTGGAT
GATCGTTGCATTCTGCACTGCGCCAACTTCAACGTGCTGTTCAGCACTGTTTTCCCGCCGACGAGCTTTGGTCCGCT
GGTCCGTAAGATTTTTGTGGATGGCGTGCCGTTCGTTGTGTCGACGGGTTATCATTTTCGTGAGCTGGGCGTTGTCC
ATAATCAGGATGTGAATCTGCATAGCAGCCGTCTGTCCTTTAAAGAACTGCTGGTCTATGCAGCGGACCCTGCAAT
GCACGCAGCAAGCGGTAATCTGCTGTTGGACAAGCGTACCACTTGCTTTAGCGTGGCTGCGCTGACCAACAATGTT
GCCTTTCAGACGGTCAAACCGGGTAACTTTAACAAGGACTTCTACGACTTTGCAGTTTCCAAGGGCTTTTTCAAAG
AGGGTTCCTCCGTTGAACTGAAACACTTCTTCTTCGCGCAAGACGGTAATGCTGCGATTAGCGATTACGATTACTA
TCGCTACAACCTGCCGACCATGTGTGATATTCGCCAGTTGCTGTTCGTAGTCGAGGTGGTCGATAAGTATTTTGATT
GCTATGACGGCGGCTGCATCAATGCGAACCAGGTGATCGTTAACAATCTGGATAAGAGCGCAGGCTTTCCGTTCAA
TAAGTGGGGCAAGGCCCGTCTGTACTATGACAGCATGAGCTATGAGGACCAAGACGCGTTGTTCGCGTACACCAA
ACGCAATGTGATTCCAACCATCACGCAAATGAATCTGAAATACGCGATCAGCGCTAAGAATCGTGCCCGCACCGT
CGCGGGTGTCAGCATCTGCAGCACCATGACCAATCGCCAGTTCCATCAGAAACTGTTGAAGAGCATTGCGGCGACT
CGCGGTGCCACGGTCGTCATTGGTACCTCTAAGTTTTATGGTGGCTGGCACAATATGTTGAAAACGGTTTACAGCG
ACGTGGAAACCCCACACCTGATGGGCTGGGATTATCCGAAATGTGACCGTGCCATGCCGAACATGCTGCGTATCAT
GGCGTCTTTGGTGCTGGCCCGTAAACATAACACCTGCTGTAATCTGAGCCACCGTTTCTACCGCTTGGCGAACGAA
TGCGCGCAAGTCCTGTCCGAGATGGTGATGTGTGGCGGCTCTCTGTACGTAAAACCGGGTGGTACCAGCAGCGGTG
ATGCAACGACCGCTTACGCTAACAGCGTGTTTAACATTTGCCAGGCGGTTACCGCAAACGTGAACGCGCTGCTGAG
CACTGATGGTAACAAAATCGCGGACAAATATGTTCGCAACCTGCAACATCGTCTGTATGAATGTCTGTACCGTAAT
CGTGACGTCGACCATGAATTTGTTGACGAGTTTTACGCGTACCTGCGCAAACATTTCAGCATGATGATCCTGAGCG
ATGACGCGGTTGTTTGCTATAACAGCAATTATGCGGCTCAGGGCCTGGTGGCATCTATCAAAAACTTCAAGGCGGT
CCTGTATTATCAGAATAATGTCTTTATGAGCGAGGCCAAGTGCTGGACGGAAACCGACCTGACTAAAGGTCCGCAT
GAGTTTTGTAGCCAACACACCATGTTGGTTAAACAAGGCGACGACTACGTGTACCTGCCGTACCCGGATCCGTCGC
GCATCCTGGGTGCAGGTTGTTTCGTGGATGACATCGTCAAGACCGATGGTACGCTGATGATCGAACGCTTCGTGTC
CCTGGCAATTGATGCCTATCCGCTGACGAAACATCCGAATCAAGAGTATGCAGATGTCTTTCACCTGTATCTGCAA
TACATTCGTAAGCTGCATGATGAGTTGACGGGTCACATGCTGGATATGTACAGCGTTATGCTGACCAATGACAACA
CCAGCCGTTACTGGGAACCGGAGTTCTATGAAGCAATGTATACCCCGCACACCGTTCTGCAGGAAAACCTGTACTT
CCAGGGTCATCACCACCATCACCATCACCATTAA

Supplementary Table 3. Codon optimised sequence of SARS-CoV nsp12 with C-terminal TEV protease cleavage site and 8-histidine tag

NCoV_FI_F1	ATTAAAGGTTTATACCTTCCCAGG
NCoV_R-1	GGGTAGAAAGAACAATACATATGTG
NCoV_FII_F-1	GCATTGATTTAGATGAGTGGAGTATG
NcoV_FII_R-1	GCAGTTAAATCCCATTTAAAAGATG
NcoV_FIII_F-1	GTGGTTTAGATTCTTTAGACACC
NCoV_FIII_R-1	GTGTCCACACTCTCCTAGCACC
NCoV_FIV_F-1	GTGTTATGTATGCATCAGCTG
NCoV_FIV_R-1	GTGGCGGCTATTGATTTCAAT
NCoV_FV_F-1	AGTACTATGACCAATAGACAGTTTC
NCoV_FV_R-1	ACTTTTATCAAAAGCTGGTGTGTGG
NCoV_FVI_F-1	GCTATCTAACCTTAACTTGCC
NCoV_FVI_R-1	GCTGGTGCATGTAGAAGTTC
NcoV_FVII_F-1	GCACACCTTGTAATGGTGTTG
NCoV_FVII_R-1	GCAAATTGTAGAAGACAAATCC
NCoV_FVIII_F-1	GCAGATTCCAACGGTACTATTACC
NCoV_FVIII_R1	GTCATTCTCCTAAGAAGCTATTAAAAT

Supplementary Table 4. Specific primers used in the sequencing study

Supplementary References

1. Subissi, L. *et al.* One severe acute respiratory syndrome coronavirus protein complex integrates processive RNA polymerase and exonuclease activities. *Proc. Natl. Acad. Sci.* **111**, E3900–E3909 (2014).
