# Supplementary material for: Rapid incorporation of Favipiravir by the fast and permissive viral RNA polymerase complex results in SARS-CoV-2 lethal mutagenesis
Source: Nat Commun. 2020 Sep 17;11:4682. doi: 10.1038/s41467-020-18463-z (PMC7499305; doi:10.1038/s41467-020-18463-z)
Supplement: Supplementary file 3 — Reporting Summary [file 41467_2020_18463_MOESM3_ESM.pdf]

nature research

Corresponding author(s): Bruno Canard and Olve Peersen

Last updated by author(s): Aug 7, 2020

Reporting Summary

Nature Research wishes to improve the reproducibility of the work that we publish. This form provides structure for consistency and transparency in reporting. For further information on Nature Research policies, see our Editorial Policies and the Editorial Policy Checklist.

Statistics

For all statistical analyses, confirm that the following items are present in the figure legend, table legend, main text, or Methods section.

n/a	Confirmed	
☐	☒	The exact sample size (*n*) for each experimental group/condition, given as a discrete number and unit of measurement
☐	☒	A statement on whether measurements were taken from distinct samples or whether the same sample was measured repeatedly
☐	☒	The statistical test(s) used AND whether they are one- or two-sided<br>*Only common tests should be described solely by name; describe more complex techniques in the Methods section.*
☒	☐	A description of all covariates tested
☒	☐	A description of any assumptions or corrections, such as tests of normality and adjustment for multiple comparisons
☒	☐	A full description of the statistical parameters including central tendency (e.g. means) or other basic estimates (e.g. regression coefficient) AND variation (e.g. standard deviation) or associated estimates of uncertainty (e.g. confidence intervals)
☒	☐	For null hypothesis testing, the test statistic (e.g. *F*, *t*, *r*) with confidence intervals, effect sizes, degrees of freedom and *P* value noted<br>*Give P values as exact values whenever suitable.*
☒	☐	For Bayesian analysis, information on the choice of priors and Markov chain Monte Carlo settings
☒	☐	For hierarchical and complex designs, identification of the appropriate level for tests and full reporting of outcomes
☒	☐	Estimates of effect sizes (e.g. Cohen's *d*, Pearson's *r*), indicating how they were calculated

Our web collection on statistics for biologists contains articles on many of the points above.

Software and code

Policy information about availability of computer code

Data collection	*Provide a description of all commercial, open source and custom code used to collect the data in this study, specifying the version used OR state that no software was used.*
Data analysis	*Provide a description of all commercial, open source and custom code used to analyse the data in this study, specifying the version used OR state that no software was used.*

For manuscripts utilizing custom algorithms or software that are central to the research but not yet described in published literature, software must be made available to editors and reviewers. We strongly encourage code deposition in a community repository (e.g. GitHub). See the Nature Research guidelines for submitting code & software for further information.

Data

Policy information about availability of data

All manuscripts must include a data availability statement. This statement should provide the following information, where applicable:

- Accession codes, unique identifiers, or web links for publicly available datasets
- A list of figures that have associated raw data
- A description of any restrictions on data availability

SRA accession number - PRJNA633443, GenBank accession numbers MT594401 and MT594402

Field-specific reporting

Please select the one below that is the best fit for your research. If you are not sure, read the appropriate sections before making your selection.

- [x] Life sciences
- [ ] Behavioural & social sciences
- [ ] Ecological, evolutionary & environmental sciences

For a reference copy of the document with all sections, see nature.com/documents/nr-reporting-summary-flat.pdf

Life sciences study design

All studies must disclose on these points even when the disclosure is negative.

Sample size	In vitro biochemistry experiments were done with three or more repeats across a range of nucleotide or analog compound concentrations to establish incorporation rates. Results were reproducible and highly consistent across data sets. Cell based infectious virus studies were repeat in triplicate.
Data exclusions	Occasional random outliers data points, but no complete datasets, were excluded from analysis. Exclusion criteria were attributed to imaging artifacts in the gel analysis and subsequent quantification.
Replication	For all quantitative data, number of replicates and SEM or SE shown in paper, with experiments run under multiple related conditions, at multiple timepoints to obtain full data sets and time curves. Results were highly reproducible, and highly consistent with different data sets
Randomization	For the most part, randomisation was not relevant to our study. For QFM measurements, potential systematic instrument error was controlled for by repeating the 100 ms reaction time at multiple points during the full 20-point data collection sequence.
Blinding	Blinding was not performed in our studies, as there was no human subjective elements in assessing experimental outcomes. Data analysis was additionally performed independantly by two persons to verify results

Reporting for specific materials, systems and methods

We require information from authors about some types of materials, experimental systems and methods used in many studies. Here, indicate whether each material, system or method listed is relevant to your study. If you are not sure if a list item applies to your research, read the appropriate section before selecting a response.

Materials & experimental systems

n/a	Involved in the study	
x		Antibodies
	x	Eukaryotic cell lines
x		Palaeontology and archaeology
x		Animals and other organisms
x		Human research participants
x		Clinical data
x		Dual use research of concern

Methods

n/a	Involved in the study	
x		ChIP-seq
x		Flow cytometry
x		MRI-based neuroimaging

Eukaryotic cell lines

Policy information about cell lines

Cell line source(s)	VeroE6 source ATCC CRL-1586
Authentication	None of the cell lines were authenticated in house; however came directly from ATCC source
Mycoplasma contamination	Confirmed tested negative for mycoplasma testing
Commonly misidentified lines (See ICLAC register)	No commonly misidentified lines according to ICLAC register
